# The Precision Paradigm in Periodontology: A Multilevel Framework for Tailored Diagnosis, Treatment, and Prevention

**DOI:** 10.3390/jpm15090440

**Published:** 2025-09-16

**Authors:** Gianna Dipalma, Angelo Michele Inchingolo, Francesco Inchingolo, Irene Palumbo, Lilla Riccaldo, Mariafrancesca Guglielmo, Roberta Morolla, Andrea Palermo, Grazia Marinelli, Alessio Danilo Inchingolo

**Affiliations:** 1 Department of Interdisciplinary Medicine, University of Bari “Aldo Moro”, 70124 Bari, Italy; gianna.dipalma@uniba.it (G.D.); angelomichele.inchingolo@uniba.it (A.M.I.); irene.palumbo@uniba.it (I.P.); lilla.riccaldo@uniba.it (L.R.); mariafrancesca.guglielmo@uniba.it (M.G.); roberta.morolla@uniba.it (R.M.); graziamarinelli@live.it (G.M.); alessiodanilo.inchingolo@uniba.it (A.D.I.); 2 Department of Biomedical, Surgical and Dental Sciences, Milan University, 20122 Milan, Italy; 3 Department of Experimental Medicine, University of Salento, 73100 Lecce, Italy; andrea.palermo@unisalento.it

**Keywords:** precision medicine, periodontology, individualized medicine, biomarkers, precision periodontology, personalized treatment

## Abstract

**Background**: Precision medicine in periodontology seeks to individualize prevention, diagnosis, and treatment based on biological, genetic, behavioral, and environmental factors. This approach addresses the limitations of standardized protocols, which often fail to consider patient-specific variability in disease susceptibility and progression. **Materials and Methods**: A systematic review was conducted following PRISMA guidelines and registered in PROSPERO (ID: CRD42024593760). Searches were performed in PubMed, Scopus, and Web of Science (2014–2025) using terms related to precision and personalized medicine in periodontology. Studies were screened based on predefined inclusion criteria, and risk of bias was assessed using the ROBINS tool. **Results**: Sixteen studies met the inclusion criteria. Diagnostic tools integrating biomarkers (e.g., IL-1β, salivary and GCF proteomics) and digital platforms (e.g., flowcharts and decision support systems) showed improved accuracy and early disease detection. Personalized treatments, including host-modulating therapies and customized antibiotics, improved clinical outcomes. Tailored preventive strategies based on genetic, systemic, and behavioral risk profiling reduced tooth loss and optimized care frequency. **Conclusions**: Precision periodontology enhances patient-centered care by integrating omics technologies, real-time diagnostics, and behavioral insights. This paradigm improves diagnostic precision, therapeutic outcomes, and long-term prevention, supporting its broader implementation in clinical practice.

## 1. Introduction

Precision medicine (PM) in periodontology represents a transformative approach aimed at tailoring the prevention, diagnosis, and treatment of periodontal diseases according to individual biological, genetic, behavioral, and environmental characteristics [1,2,3,4,5]. Traditional periodontal care has long relied on generalized clinical protocols and empirical treatments, yet mounting evidence reveals significant inter-individual variability in disease progression, microbial composition, host response (HR), and treatment outcomes, underscoring the limitations of uniform strategies [6,7,8,9,10,11]. Precision periodontics (PP) seeks to shift from a “one-size-fits-all” model to a personalized paradigm that integrates advanced diagnostics, biomarker (BM) profiling, machine learning, and tailored interventions [12,13,14,15,16].

Periodontal disease (PD)—especially periodontitis—is a complex, multifactorial chronic condition driven by dysbiotic subgingival biofilms and dysregulated host immune/inflammatory responses. Key contributors include genetic susceptibility, epigenetic regulation, systemic health factors (e.g., diabetes, cardiovascular disease), lifestyle behaviors (such as smoking and oral hygiene habits), and psychosocial elements. The interaction among these variables leads to heterogeneous clinical presentations and unpredictable therapy responses, emphasizing the need for precision strategies [17,18,19,20,21,22].

Broadly, the precision periodontics framework can be conceptualized across three interconnected pillars: precision diagnosis, personalized treatment, and individualized prevention, supported by digital integration and systems-level understanding [23,24,25,26,27,28,29,30].

### 1.1. Precision Diagnosis

Advances in omics technologies, genomics, transcriptomics, proteomics, and metabolomics, allow for the identification of specific BMs that can detect disease onset, classify disease phenotypes or subtypes, and predict progression trajectories [31,32,33,34,35,36,37]. For example, the diagnostic accuracy of microbial, salivary and subgingival BMs such as *Porphyromonas gingivalis*, IL1β, MMP8, and combined multi-marker panels has shown sensitivities and specificities exceeding 85–90% in meta-analyses [38,39,40,41,42].

Similarly, molecular markers, including proinflammatory cytokines, collagenases, and proteases, measured in saliva or gingival crevicular fluid (GCF) are highly correlated with disease severity indices such as probing depth and clinical attachment loss [43,44,45,46,47]. Recent reviews propose cumulative risk models combining microbial and cytokine markers for individualized disease prediction [48,49,50,51,52,53].

### 1.2. Personalized Treatment

Once patient-specific BMs and risk profiles are established, targeted therapeutic strategies become feasible [54,55,56,57,58,59]. These include antibiotic selection based on individual biofilm susceptibility, immunomodulatory adjunctive therapies, and HR modulation [60,61,62,63,64]. Customized antibiotic regimens guided by real-time biofilm susceptibility testing have been shown to yield superior reduction in pathogenic bacteria and better clinical outcomes compared to conventional methods [65,66,67,68,69]. Similarly, adjunctive technologies such as laser therapy, ozone, or advanced antimicrobial scaffolds offer multimodal mechanisms—biofilm disruption plus host modulation—that can be harnessed in tailored protocols [70,71,72,73].

Machine learning-enhanced prediction tools have also enabled stratification of patients into responders vs. non-responders for various nonsurgical treatments [74,75,76,77,78]. Moreover, intelligent algorithms trained on clinical, genetic, and imaging data can optimize the timing and combination of interventions according to patient-specific trajectories [79,80,81,82,83,84].

### 1.3. Individualized Prevention

Beyond clinical treatment, PP emphasizes personalized preventive strategies. Identification of behavioral, systemic, and environmental risk factors facilitates the design of patient-centered educational and motivational interventions [85,86,87,88,89]. Evidence shows that tailored communication, goal setting, and coaching significantly improve long-term plaque control and gingival health [90,91,92,93,94,95,96]. Integrating risk profiling approaches borrowed from chronic disease domains—such as rheumatoid arthritis education models—into periodontology can improve patient engagement and adherence [97,98,99,100,101].

Stratified preventive schedules—adjusting recall intervals, hygiene reinforcement, and professional debridement based on individualized risk—have demonstrated improved outcomes and cost-efficiency in long-term cohorts [102,103,104,105]. Approaches that combine genotypic, metabolic, and behavioral risk factors to determine frequency of recall visits can reduce tooth loss in high-risk patients, while avoiding overtreatment in low-risk individuals [106,107,108,109,110].

### 1.4. Integration of Digital Tools and Systems Biology

The implementation of PP is underpinned by digital infrastructure such as electronic dental records (EDR), decision support systems, and point-of-care diagnostics [111,112,113]. These enable real-time risk assessment based on multifactorial data, facilitate clinician–patient communication, and support evidence-based personalized planning [114,115]. In parallel, systems biology approaches and multi-omics data integration—via machine learning and network analysis—unravel molecular pathways linking PD to systemic disorders and suggest novel therapeutic targets [116,117,118,119].

PD exhibits strong associations with systemic conditions such as cardiovascular disease, diabetes, rheumatoid arthritis, and adverse pregnancy outcomes. PM strategies that identify shared genetic or inflammatory BMs may guide interventions addressing both oral and systemic health [120,121].

### 1.5. Educational and Clinical Workflow Adaptation

Embedding precision paradigms into clinical education and workflows involves developing user-friendly tools such as diagnostic flow-charts, risk stratification flow panels, and clinician support apps. These tools help standardize diagnostic classifications, increase diagnostic confidence, and reduce variability across clinicians, especially for early-career professionals.

In summary, PM in periodontology is a multidimensional model built upon diagnostic precision, targeted personalized therapy, and individualized prevention, all integrated via digital and behavioral frameworks. This paradigm empowers clinicians to

- Detect PD earlier and more accurately via sensitive BMs;
- Customize treatment according to patient-specific characteristics and biofilm susceptibility;
- Drive preventive strategies based on individualized risk profiles and behavioral insights;
- Improve clinician–patient engagement using clear risk communication and educational tools;
- Enhance efficiency and sustainability of care by optimizing recall intervals and resource allocation.

This systematic review aims to explore the current landscape of PM in periodontology, with a specific focus on the integration of digital diagnostic tools, molecular BMs, and personalized preventive strategies to enhance patient-centered care and clinical outcomes.

## 2. Materials and Methods

### 2.1. Protocol and Registration

The protocol was registered at PROSPERO with the ID CRD42024593760, and the systematic review was carried out in accordance with the Preferred Reporting Items for Systematic Reviews and Meta-Analyses (PRISMA) guidelines.

### 2.2. Search Processing

Using PubMed, Scopus, and Web of Science, articles about precision medicine and its use in periodontology have been identified between 1 January 2014 and 30 May 2025. (“personalized medicine” OR “precision medicine” OR “personalized medicine” OR “individualized medicine” OR “stratified medicine”) AND (“periodontics” OR “periodontology” OR “periodontitis” OR “periodontal disease” OR “periodontal health” OR “gum disease”). For this review, only peer-reviewed articles were included to ensure methodological rigor and sufficient reporting of outcomes. Gray literature, such as conference abstracts, dissertations, and non-peer-reviewed reports, was not considered due to limited availability of detailed methodological information and potential variability in quality.

### 2.3. Inclusion Criteria

The inclusion criteria were as follows: (1) papers that investigated personalized medicine in relation to periodontal disease; (2) they were designed as randomized clinical trials, retrospective studies, case–control studies, case series, case reports, or prospective studies; (3) they were published in English; and (4) the full text was available.

Papers that did not match the above criteria were excluded.

The review was conducted using the following PICOS criteria:

Participants: Patients with periodontal diseases.
Interventions: Precision medicine approaches (personalized diagnostics, treatments, and prevention).
Comparisons: Standard periodontal care.
Outcomes: Diagnostic accuracy, treatment effectiveness, and prevention efficacy.
Study: Clinical studies (RCTs, cohort, case–control, cross-sectional, experimental, and bioinformatic).

### 2.4. Exclusion Criteria

The exclusion criteria were as follows: (1) study involving animals; (2) irrelevant subjects; (3) reviews, letters, or comments; and (4) non-English language.

### 2.5. Data Processing

After conducting independent database searches, three reviewers (M.G., I.P., and R.M.) assessed the studies’ quality using the selection criteria. Version 6.0.15 (Corporation for Digital Scholarship, Vienna, VA, USA) was used to download the chosen articles. A senior reviewer (F.I.) was consulted to resolve any disagreements amongst the three writers.

### 2.6. Quality Assessment

The quality of the included papers was assessed by three reviewers—M.G., I.P., and R.M.—using the ROBINS tool (Version 2022—Cochrane Bias Methods Group, London, UK).

Each of the seven points under evaluation had a bias level assigned to it. A third reviewer (F.I.) was consulted in cases of disagreement until an agreement was reached. Among the domains evaluated by ROBINS were the following:

- Confounding bias;
- Bias resulting from exposure measurement;
- Bias in a study’s participant selection;
- Bias resulting from post-exposure intervention;
- Bias resulting from missing data;
- Bias resulting from outcome measurement,
- Bias in the presentation of the results.

## 3. Results

### 3.1. Study Selection

A total of 854 articles were identified through the electronic database search (275 from Scopus, 373 from PubMed, and 206 from Web of Science), while no additional studies were found via manual searching.

After removing 125 duplicates, 729 titles and abstracts were screened. Of these, 63 were selected for full-text review because 666 were excluded (581 were unrelated to the topic, 84 were review articles, and 1 was an animal study). Following full-text assessment, 47 studies were excluded (37 were off-topic and 10 were reviews), resulting in 16 articles being included in the qualitative analysis.

**Figure 1:**
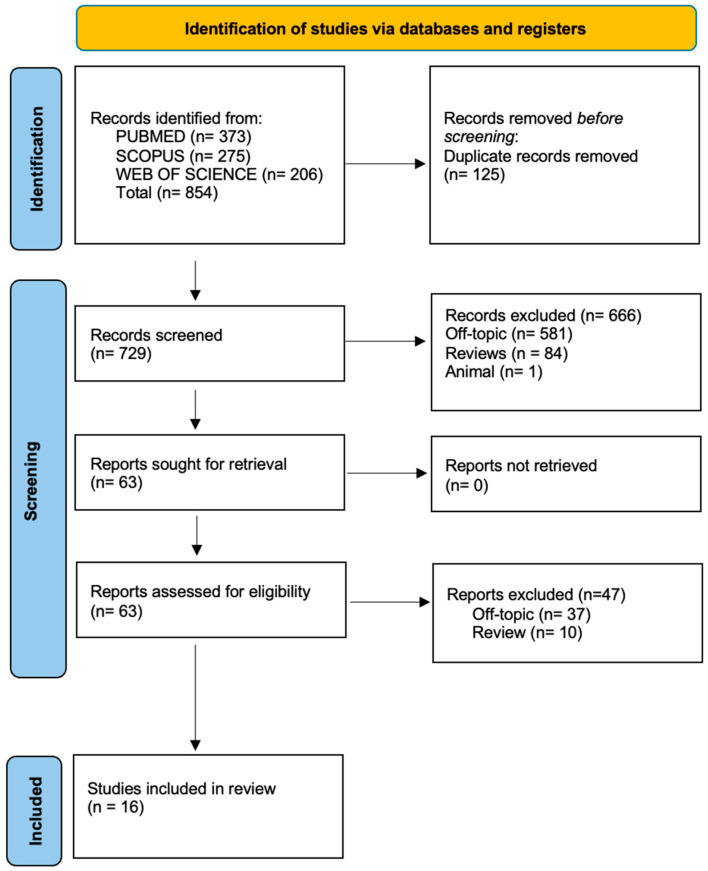
Literature search following the Preferred Reporting Items for Systematic Reviews and Meta-Analyses (PRISMA) flow diagram and database search indicators.

The selection process is illustrated in Figure 1, and the included studies are summarized in Table 1.

### 3.2. Quality Assessment and Risk of Bias of Included Articles

**Figure 2:**
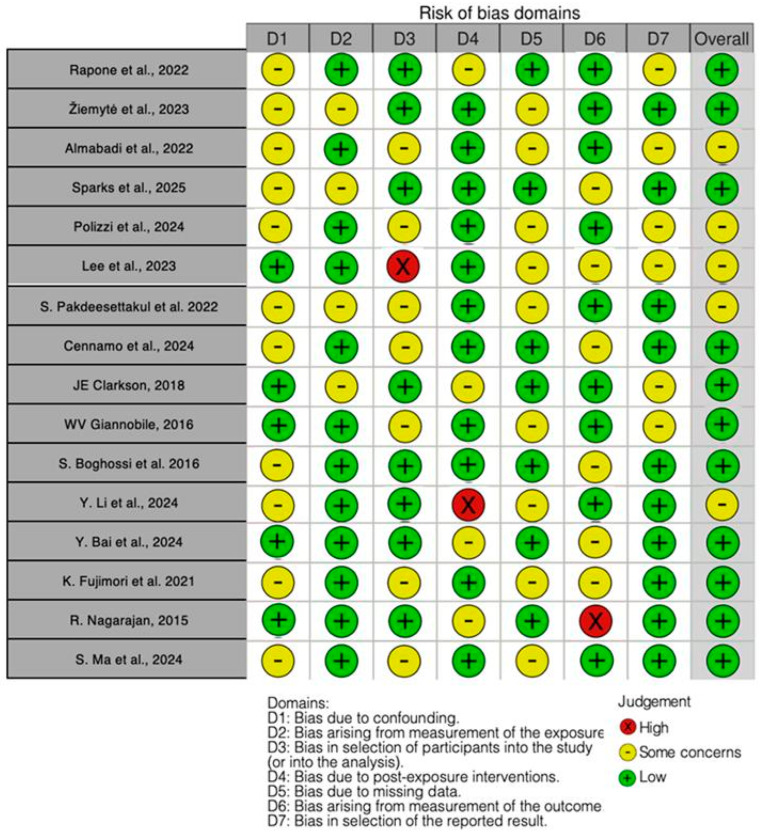
Bias assessment. The figure highlights the proportion of studies categorized as having low, high, or unclear risk of bias in each domain, providing a visual summary of the overall methodological quality of the included studies. Rapone et al., 2022 [122]; Žiemytė et al., 2023 [123]; Almabadi et al., 2022 [124]; Sparks et al., 2025 [125]; Polizzi et al., 2024 [126]; Lee et al., 2023 [127]; S. Pakdeesettakul et al., 2022 [128]; Cennamo et al., 2024 [129]; JE Clarkson, 2018 [130]; WV Giannobile, 2016 [131]; S. Boghossi et al., 2016 [132]; Y. Li et al., 2024 [133]; Y. Bai et al., 2024 [134]; K. Fujimori et al., 2021 [135]; R. Nagarajan, 2015 [136]; S. Ma et al., 2024 [137].

Regarding the bias due to confounding, most studies present some concerns, with 11 studies categorized in this group, while 5 studies have a low risk. The bias arising from measurement of the exposure is generally a parameter with a low risk of bias, as 12 studies fall into this category, while 4 show some concerns. Many studies exhibit a low risk of bias in the selection of participants, with 8 studies classified as low risk, 7 showing some concerns, and only 1 study presenting a high risk. Bias due to post-exposure interventions shows 11 studies rated as low risk, 4 with some concerns, and 1 with high risk. The bias due to missing data is generally a parameter with a low risk of bias, as 7 studies fall into this category, while 9 show some concerns. Bias arising from measurement of the outcome is also low, with 9 studies categorized as low risk, 6 with some concerns, and 1 as high risk. Regarding bias in the selection of the reported studies, 10 have some concerns, while 6 have a low risk (Figure 2).

## 4. Discussion

### 4.1. Advances and Integration of Precision Medicine Approaches in Periodontology

PM in periodontology aims to tailor treatments based on individual HR, genetic predisposition, and microbial profiles. Recent advances illustrate how personalized approaches can optimize both therapy and prevention in periodontal care by targeting the multifactorial nature of the disease.

Rapone et al. suggest that adjunctive therapies such as gaseous ozone can be incorporated into personalized treatment protocols to enhance periodontal healing. Ozone’s immunomodulatory, antimicrobial, antihypoxic, and biosynthetic properties contribute to improved clinical outcomes, as demonstrated in their randomized controlled trial, where scaling and root planing combined with ozone therapy significantly improved probing pocket depth, clinical attachment level, and bleeding on probing compared to conventional therapy alone. This dual mechanism—biofilm reduction and modulation of host inflammatory response—highlights ozone’s promise in both therapeutic and preventive contexts within precision periodontology [122].

Similarly, Žiemytė et al. (2023) [123] developed an innovative impedance-based real-time biofilm growth monitoring system to personalize antibiotic selection. Their double-blind randomized controlled trial demonstrated that antibiotics chosen by this ex vivo biofilm susceptibility testing led to greater reductions in pathogenic bacteria and plaque, surpassing traditional hybridization-based selection. This strategy addresses limitations of standard molecular methods by evaluating the entire subgingival biofilm’s antibiotic susceptibility quickly and cost-effectively, offering a targeted antimicrobial approach that can minimize disease recurrence through precision microbial management. Žiemytė et al. [123] provided another example of precision intervention through ex vivo biofilm susceptibility testing. By tailoring antibiotic selection to the actual biofilm composition, they achieved superior microbial control compared with traditional methods. This approach directly addresses the global issue of antibiotic resistance, but its feasibility and cost-effectiveness in daily clinical settings remain to be proven.

Beyond therapeutic interventions, prevention emerges as a critical component of PM. Prevention through behavioral modification is equally critical. Almabadi et al. (2021) [124] evaluated a personalized oral health education program employing tailored messages, goal-setting, and motivational interviewing. Their trial showed significant improvements in plaque control and gingival inflammation. This reinforces the idea that PM is not limited to molecular diagnostics but must also include behavioral precision, addressing patient adherence as a central determinant of success, demonstrating that personalized behavioral interventions can effectively augment preventive outcomes. These findings reinforce the role of precision prevention, where individualized education and motivation complement clinical therapies to reduce disease progression.

From a broader perspective, Sparks et al. emphasize the importance of integrating genetic, environmental, and behavioral risk factors into personalized risk communication. Their PRE-RA study on rheumatoid arthritis illustrates how tailored education enhances risk awareness and promotes preventive behaviors. This model is highly relevant to periodontology, where similar complexities of host susceptibility and environmental triggers exist. Identification of patient-specific risk profiles—including smoking status and microbial dysbiosis—can guide personalized prevention and early intervention, fostering improved adherence and clinical outcomes. Their work calls for further validation of BMs and development of practical tools to support personalized risk assessment in periodontal care [125].

At the molecular level, Polizzi et al. investigated the role of microRNAs (miRNAs) in alveolar bone remodeling during orthodontic tooth movement through a randomized clinical trial. By analyzing miRNA expression in gingival crevicular fluid, they identified specific miRNAs as BMs of bone resorption and formation. These insights offer a molecular basis for personalizing orthodontic force application, enhancing treatment efficacy while minimizing periodontal side effects. This exemplifies how molecular profiling can refine precision therapies not only in orthodontics but also in periodontology, facilitating biologically informed and individualized clinical protocols [126].

Together, these studies underscore the multidimensional nature of PM in periodontology—integrating adjunctive antimicrobial therapies, innovative diagnostic tools, personalized behavioral interventions, genetic and environmental risk profiling, and molecular BMs. The future of periodontal care lies in harnessing such personalized approaches to optimize prevention and treatment tailored to each patient’s unique biological and behavioral context, ultimately improving long-term outcomes and reducing disease burden.

### 4.2. Integrating Precision Diagnostics into Periodontal Practice: Clinical Tools and Molecular Innovations

Recent literature highlights a progressive shift toward the clinical integration of PM in periodontology, particularly in the diagnostic domain, where the goal is to deliver more accurate, personalized, and timely identification of PD. The study by Lee (2024) [127] marks a significant advancement in this direction through the development and clinical implementation of the Precision Periodontal Health Care Chart (PPHCC), a digital tool embedded within the EDR system. This platform consolidates periodontal clinical parameters—such as probing depth (PD), bleeding on probing (BOP), plaque index, and bone loss—with systemic comorbidities like diabetes and cardiovascular disease, thereby generating individualized risk profiles based on a validated Precision Periodontal Risk Assessment (PPRA) model. Although no statistically significant short-term differences in clinical parameters were observed when compared to standard care, the PPHCC improved diagnostic communication, patient awareness, and engagement with care plans, representing a foundational step in the patient-centered application of diagnostic precision. Although initial findings suggest improvements in patient communication, the absence of significant clinical outcome differences raises the question of whether digital risk tools can translate into tangible health gains without long-term validation.

In parallel, the randomized crossover study conducted by Pakdeesettakul et al. (2022) [128] further supports the adoption of simplified diagnostic tools by demonstrating that the use of structured diagnostic flowcharts, grounded in the 2018 periodontal classification, significantly enhanced diagnostic accuracy and clinician self-confidence, particularly among less experienced practitioners. While expert clinicians maintained high diagnostic accuracy regardless of the tool used, the structured nature of the flowcharts facilitated more consistent staging, grading, and extent classification, without increasing diagnostic time. This highlights the importance of standardized yet flexible tools that improve decision-making across all levels of clinical experience, aligning with the core objectives of PM to reduce variability and optimize individual care pathways. Pakdeesettakul et al. demonstrated that structured diagnostic flowcharts improve accuracy, particularly for less experienced clinicians. This highlights a potential role for PM tools in reducing inter-operator variability, a persistent challenge in periodontal diagnosis. However, the reliance on flowcharts risks oversimplifying complex patient profiles if not integrated with biological or behavioral data.

In addition to these decision-support tools, molecular diagnostics have emerged as a critical component of PP: The study by Cennamo et al. (2024) [129] introduced an innovative surface plasmon resonance–plastic optical fiber (SPR-POF) biosensor designed for the rapid, label-free detection of interleukin-1β (IL-1β) in saliva, a key proinflammatory BMs associated with the early phases of periodontitis. The biosensor demonstrated high specificity, low detection limits (15.5 pM in buffer and 23.4 pM in saliva), and accurate discrimination between periodontal health and disease. Importantly, it offers real-time diagnostic results within minutes, showing strong agreement with standard ELISA assays. This non-invasive, point-of-care device represents a promising leap forward in enabling early and individualized diagnosis—potentially identifying disease activity prior to the appearance of clinical symptoms—thereby allowing for more timely and targeted interventions. biosensor technologies such as the SPR-POF device by Cennamo et al. [129] represent a major advance, allowing rapid and non-invasive detection of IL-1β. Such innovations move PM closer to point-of-care reality, but most remain prototypes and lack large-scale clinical validation.

Complementing these findings, Silva-Boghossian et al. (2013) [132] explored the diagnostic potential of proteomic analysis of GCF in differentiating periodontal health, gingivitis, and periodontitis. Through mass spectrometry-based proteomic profiling, the study identified distinct expression patterns of key inflammatory and immune-related proteins—including S100A8, S100A9, and azurocidin—that were elevated in diseased states but minimal or absent in health. This BMs-driven approach underscores the potential of GCF proteomics to serve as a robust platform for early-stage diagnosis and disease monitoring, reinforcing the role of biochemical individuality in periodontal assessment.

Collectively, these studies demonstrate the multifaceted nature of precision diagnostics in periodontology, encompassing both technological innovations and molecular profiling. From digital risk assessment tools and diagnostic flowcharts that guide clinical reasoning, to biosensors and proteomics that detect disease at the molecular level, the integration of PM offers a more holistic, patient-tailored approach to periodontal care. These tools not only enhance diagnostic accuracy and clinician confidence but also improve patient involvement and open new avenues for early intervention. As the field moves forward, further research will be essential to validate these approaches in diverse populations and to determine their long-term impact on periodontal health outcomes. Nonetheless, the current evidence strongly supports the paradigm shift toward precision diagnostics as a cornerstone of future periodontal practice. In summary, precision diagnostics in periodontology currently spans a spectrum from digital flowcharts to high-throughput proteomics. The key challenge is bridging this technological diversity with clinical usability, cost-effectiveness, and integration into daily practice.

### 4.3. Implementing Precision Prevention in Periodontology: Evidence for Risk-Based and Personalized Care Strategies

The integration of PM into preventive periodontal care is exemplified by both the IQuaD trial and the retrospective cohort study by Giannobile et al., which collectively underscore the clinical and economic benefits of patient stratification in guiding personalized interventions. The IQuaD trial, a large multicenter randomized controlled study, demonstrated that individualized oral hygiene advice (OHA), grounded in behavioral science, and tailored periodontal instrumentation (PI) schedules can improve clinical outcomes such as gingival inflammation and promote greater patient self-efficacy. By considering patient-specific periodontal status and behavioral readiness, the study highlighted how customized action plans can enhance engagement and potentially reduce disease burden when integrated into routine dental care [130]. Complementing these findings, Giannobile’s long-term cohort study provided compelling evidence for risk-based prevention by stratifying patients according to genetic (IL-1 genotype), metabolic (diabetes), and behavioral (smoking) risk factors. The results showed that high-risk individuals benefited significantly from biannual preventive visits, with reduced rates of tooth loss over a 16-year period, while low-risk patients did not experience a comparable advantage from more frequent visits [131].

These outcomes collectively reinforce the rationale for shifting from a uniform model of preventive care to one that is risk-adapted and personalized, thereby aligning preventive strategies with individual susceptibility. Such an approach not only enhances clinical effectiveness but also promotes resource efficiency and long-term oral health sustainability, establishing a strong foundation for the widespread adoption of precision prevention in dental practice.

### 4.4. Multi-Omics Discovery of Immune and Genetic Biomarkers in Periodontitis

The evolving landscape of periodontology has increasingly embraced the principles of PM, with recent research focusing on dissecting the molecular and immunological complexity of periodontitis. By leveraging multi-omics approaches, machine learning, and systems biology, several studies have illuminated novel molecular signatures, patient-specific BMs, and disease subtypes that pave the way for more individualized diagnostic and therapeutic strategies. The reviewed omics studies illustrate the potential of computational and molecular tools to identify patient-specific biomarkers and molecular subtypes of periodontitis.

This represents a critical step toward stratifying patients not just by clinical phenotype but by underlying pathophysiology. For instance, Li et al. identified immune-related genes with causal roles in periodontitis, while Bai et al. and Fujimori et al. validated hub genes associated with immune cell infiltration [133,134,135]. These findings open the possibility of molecularly defined disease subtypes.

Y. Li et al. (2024) [133] made a substantial contribution by integrating transcriptomic data, machine learning algorithms, Mendelian randomization and single-cell RNA sequencing to identify immune-related BMs with causal roles in periodontitis. Through the analysis of differentially expressed genes (DEGs) and the application of weighted gene co-expression network analysis (WGCNA), the authors identified 471 candidate genes, narrowing them down to 19 core genes using ML methods—among which CD93, CD69, and CXCL6 demonstrated the highest diagnostic accuracy and were validated as causally linked to the disease via MR. These genes were shown to have distinct expression patterns in immune and epithelial cell types and were strongly associated with immune cell infiltration in diseased periodontal tissues. This study underscores the power of integrating computational and high-throughput biological tools to uncover clinically actionable BMs for precision diagnostics.

Building on a similar methodological foundation, Y. Bai et al. (2024) [134] utilized transcriptomic data from the GSE10334 dataset to explore gene modules involved in periodontitis. Through WGCNA, they identified 12 gene modules, with the turquoise module showing the most significant correlation with clinical disease phenotypes. Enrichment analyses revealed that these modules were predominantly involved in immune responses, cytokine signaling, and leukocyte migration—processes central to periodontal inflammation. Notably, hub genes such as PLEK, TYROBP, and LAPTM5, identified through protein–protein interaction (PPI) networks, were validated in external datasets and linked to macrophage and neutrophil activity. This integrative bioinformatics approach strongly supports the use of immune-related gene signatures as early BMs for disease detection and personalized therapeutic decision-making.

K. Fujimori et al. (2021) [135] further extended the application of integrative bioinformatics by analyzing two large-scale transcriptomic datasets (GSE10334 and GSE16134). Their WGCNA revealed that the green-yellow module was most significantly correlated with periodontitis and enriched in immune and inflammatory pathways, including cytokine–cytokine receptor interactions. Thirteen hub genes were validated via PPI and receiver operating characteristic (ROC) analysis, confirming their diagnostic relevance. Furthermore, the study established associations between gene expression patterns and specific immune cell infiltrates, such as M0 macrophages and activated mast cells. This reinforced the role of immune microenvironment modulation in the pathogenesis of periodontitis and emphasized how multi-omics and systems biology can identify clinically relevant molecular targets.

Similarly, Ma et al. [137] described four distinct molecular subtypes with unique immunometabolic profiles, suggesting that treatment may one day be tailored not just to disease severity but to molecular phenotype [137].

Adding a patient-level dimension to this molecular landscape, R. Nagarajan et al. (2015) [136] investigated inter-individual variations in salivary and serum inflammatory BMs across periodontal health, gingivitis, and periodontitis. By applying statistical clustering to clinical and molecular data, the authors demonstrated that BM trajectories varied significantly among individuals. These personalized patterns, which correlated with clinical indices such as probing depth and attachment loss, suggest that disease progression follows non-uniform molecular pathways. Their findings emphasize the importance of integrating individualized BM profiles into diagnostic protocols, enabling more accurate disease monitoring and tailored treatment strategies.

Finally, S. Ma et al. (2024) [137] employed a combined single-cell RNA sequencing and bulk transcriptome analysis to explore the molecular heterogeneity of periodontitis at a cellular level. Their analysis identified four distinct molecular subtypes: quiescent, macrophage-dominant, mitochondria-dominant, and mixed. Each subtype exhibited unique immunometabolic gene expression patterns. Through machine learning, 13 candidate BMs were identified, and five (BNIP3, FAHD1, UNG, CBR3, and SLC25A43) were validated in clinical samples, with BNIP3, FAHD1, and UNG significantly downregulated in diseased tissue. These BMs were particularly relevant to mitochondrial dysfunction and immune regulation, suggesting their potential utility in defining disease subtypes and developing precision-targeted therapies.

Together, these five studies offer a compelling synthesis of how PM can reshape the future of periodontology. By uncovering molecular subtypes, identifying causative BMs, and validating diagnostic tools at both the population and individual level, they collectively highlight the transition from generalized clinical frameworks to a model of care grounded in molecular specificity. Future directions should focus on the clinical translation of these findings, including the development of accessible diagnostic platforms, integration with electronic health records, and the design of personalized intervention protocols. The convergence of omics technologies and computational biology stands to revolutionize the prevention, diagnosis, and treatment of PD, aligning periodontology with the broader movement toward individualized healthcare.

These studies collectively demonstrate the transformative potential of precision medicine in periodontology, shifting care from generalized protocols to approaches based on molecular specificity. By identifying disease subtypes, validating biomarkers, and advancing diagnostic tools, they pave the way for more individualized strategies. Future efforts should prioritize clinical translation through accessible diagnostic platforms, integration with digital health systems, and personalized interventions, positioning periodontology within the broader evolution of precision healthcare.

## 5. Strengths and Limitations

This systematic review presents several strengths. First, it addresses a novel and highly relevant topic by exploring the applications of precision medicine in periodontology, an emerging field with substantial potential to transform clinical practice. Second, the review integrates evidence from both clinical studies and bioinformatic/multi-omics research, providing a broader and more comprehensive perspective compared to previous reviews that typically focused on only one dimension. Third, the inclusion of recent and up-to-date references, the majority published within the last five years, ensures that the findings reflect the most current scientific advances. Finally, the methodological rigor adopted in study selection and synthesis enhances the reliability and reproducibility of the review.

Despite providing a comprehensive overview of current applications of precision medicine (PM) in periodontology, this systematic review presents several limitations. First, heterogeneity among included studies—in terms of study design, sample size, follow-up duration, and diagnostic methodologies—limits direct comparison and meta-analytic synthesis. Second, many of the included trials are early-phase or exploratory, lacking long-term clinical validation or standardization of outcomes. Third, there is a geographical bias, with most studies originating from high-income countries, potentially limiting generalizability to broader populations. Moreover, the variability in biomarker selection and measurement platforms complicates the reproducibility and clinical translation of results. Lastly, the integration of digital tools and AI-driven diagnostics, though promising, remains at a conceptual or prototype level in most studies, requiring further real-world testing to establish feasibility, cost-effectiveness, and clinician acceptance.

## 6. Conclusions

The application of precision medicine (PM) in periodontology marks a pivotal evolution in the prevention, diagnosis, and treatment of periodontal disease (PD). Through the integration of host response (HR) profiling, genetic and microbial analyses, and behavioral risk assessment, PM enables individualized clinical decision-making that transcends traditional, uniform protocols. Evidence from randomized controlled trials and bioinformatics-driven studies supports the clinical validity of diverse PM approaches—from adjunctive therapies like ozone and biofilm-guided antibiotic selection, to digital diagnostics and salivary or gingival crevicular fluid (GCF) biomarker profiling. The incorporation of machine learning, multi-omics, and electronic dental records (EDR) has fostered a deeper understanding of disease heterogeneity, while also enhancing diagnostic precision and therapeutic predictability. Studies evaluating tools such as the Precision Periodontal Health Care Chart (PPHCC), structured diagnostic flowcharts, and biosensors for IL-1β demonstrate that technology-driven diagnostics improve not only accuracy but also patient engagement and adherence.

Moreover, personalized prevention strategies grounded in risk stratification—such as tailored recall intervals and behavioral interventions—have proven both clinically effective and resource-efficient. Taken together, these findings consolidate PM as the cornerstone of future periodontal practice, offering a scalable framework for biologically informed, patient-centered care. Further longitudinal validation will be essential to support widespread clinical adoption and long-term sustainability.

## Figures and Tables

**Table 1:** Descriptive summary of item selection.

Author (Year)	Study Design	Number of Patients	Average Age & Gender	Materials and Methods	Outcomes
Rapone et al. (2022) [122]	Randomized Controlled Clinical Trial	90 patients (45 in test group, 45 in control group)	Test group (SRP + ozone): 51.62 ± 9.56 years, 87% M, 13% F; Control group (SRP): 49.88 ± 10.54 years, 78% M, 22% F	Test group received SRP plus gaseous ozone therapy (ozonated water rinses and ozone gas applications in three steps), while control group received SRP alone. Clinical parameters (PPD, CAL, BOP) assessed at baseline, 3 months, and 6 months.	The test group showed significant improvements in PPD, CAL, and BOP at both 3 and 6 months compared to SRP alone (*p* < 0.0001), indicating enhanced periodontal healing and immune modulation with adjunctive ozone therapy.
Žiemytė et al. (2023) [123]	Double-blind Randomized Controlled Trial	64 patients (32 per group)	Adults 40–70 years	Compared antibiotics selected by standard DNA hybridization vs. impedance-based biofilm culture system.	The impedance-based group had greater plaque reduction, reduction in periodontal pathogens, and increase in health-associated bacteria, enabling faster personalized therapy selection.
Almabadi et al. (2021) [124]	Randomized Controlled Trial	233 patients (117 intervention, 116 control)	Adults aged 18–60; majority female	Compared personalized oral health education (tailored messages + motivational interviewing) vs. standard care.	Intervention group showed significant reduction in plaque, gingival inflammation, and bleeding on probing at 12 months vs. control.
Sparks et al. (2015) [125]	Randomized controlled trial	Unspecified	Adult first-degree relatives; both genders included	Randomized adults with family history of RA to personalized risk education or standard information; follow-up at 6 and 12 months	Changes in risk perception and preventive behaviors after personalized education
Polizzi et al. (2024) [126]	Randomized clinical trial	Unspecified	Unspecified	Patients under orthodontic treatment randomized; gingival crevicular fluid collected; miRNA levels analyzed by qRT-PCR during tooth movement	Identification of miRNAs involved in alveolar bone remodeling; potential biomarkers for personalized orthodontic therapy
Lee et al. (2024) [127]	Prospective cohort study	26	Unspecified	The study tested the PPHCC, a digital tool for personalized periodontal diagnosis. Patients received treatment and follow-up, while usability was evaluated by both patients and providers.	The PPHCC showed high usability and improved patient engagement and communication, though short-term clinical outcomes were similar to the control group.
Pakdeesettakul et al. (2022) [128]	Randomized cross-over controlled trial	153	Approximately 60% female; most participants aged 21–25	The study compared diagnostic flowcharts to 2018 consensus reports using 25 validated cases. Participants with different experience levels evaluated cases in two sessions. The study measured diagnostic accuracy, time, confidence, and user perception.	Flowcharts improved diagnostic accuracy and self-confidence, particularly among less experienced users, without increasing diagnosis time. They were viewed as simple, useful, and preferred for clinical use.
Cennamo et al. (2024) [129]	Experimental study;	2 (1 healthy, 1 with periodontitis) + 1 healthy volunteer for calibration	Males aged 61 and 69	A SPR-POF biosensor coated with anti-IL-1β antibodies was developed and validated for detecting IL-1β in buffer and saliva. It showed good sensitivity, specificity, and stability, with dose–response curves comparable to ELISA, confirming its reliability for salivary biomarker analysis.	The biosensor accurately detected IL-1β with high sensitivity and specificity, effectively distinguishing health from disease. Its performance, comparable to ELISA, supports its use as a robust point-of-care tool for early, personalized diagnosis of periodontitis.
Clarkson et al. (2018) [130]	Pragmatic multi-centre randomized controlled trial with a factorial design	1860 patients	Unspecified	The study tested personalized oral hygiene advice and different periodontal cleaning intervals in a randomized trial, collecting clinical, patient-reported, and economic data over three years. Results aimed to identify effective, cost-efficient strategies for periodontal disease prevention.	The study found that personalized oral hygiene advice, especially when combined with tailored periodontal care, may enhance clinical outcomes and patient behaviors, offering a potentially cost-effective approach to preventing periodontal disease.
WV Giannobile et al. (2026) [131]	Retrospective cohort study	5117 genotyped participants (from 25,452 eligible)	Mean age 47 years; ~65% female	Participants were classified as low- or high-risk based on smoking, diabetes, and IL-1 genotype. Over 16 years, tooth loss and dental care costs were analyzed in relation to preventive visit frequency using insurance data and stratified statistical models.	Two annual visits significantly reduced tooth loss in high-risk patients, while no benefit was seen in low-risk patients. Personalized prevention based on risk stratification proved more effective than uniform care.
Silva-Boghossian et al. (2016) [132]	Cross-sectional proteomic study	30	Unspecified	GCF samples from subjects with health, gingivitis, or periodontitis were analyzed via LC-MS/MS to detect differences in protein expression.	Inflammatory proteins were elevated in disease, distinguishing each condition and highlighting GCF proteomics as a promising diagnostic tool for precision periodontology
Y. Li et al. (2024) [133]	Bioinformatic study integrating multi-omics, ML, MR & scRNA-seq	Unspecified	Unspecified	Transcriptomic datasets; WGCNA; ML (XGBoost); Mendelian randomization; single-cell RNA-seq	Identified 19 core immune-related genes; CD93, CD69, and CXCL6 confirmed as causal genes in periodontitis. Precision medicine validated for biomarker discovery and stratification.
Y. Bai et al. (2024) [134]	Transcriptomic and network-based bioinformatic analysis	Unspecified	Unspecified	GSE10334 dataset; WGCNA; GO/KEGG pathway enrichment; PPI network; external validation datasets	12 gene modules identified; PLEK, TYROBP, LAPTM5 selected as hub genes. Pathways related to immune response and leukocyte migration highlighted.
Fujimori et al. (2021) [135]	Integrative transcriptomic study using two datasets	44 patients	median age, 67.1 years	GSE10334 and GSE16134 datasets; WGCNA; PPI network; ROC curve analysis; immune cell infiltration profiling	Green-yellow gene module significantly associated with periodontitis; 13 hub genes validated; macrophages and mast cells linked to disease pathogenesis.
Nagarajan et al. (2015) [136]	Observational cross-sectional study with statistical clustering	100 (approx.)	Mixed; age ~varied (NR)	Clinical cohort from University of Kentucky; saliva and serum biomarker analysis; cytokines and chemokines; unsupervised clustering; statistical modeling	Biomarker variation shown across individuals; transitions from health to disease are patient-specific. Supports personalized monitoring using inflammatory profiles.
S. Ma et al. (2024) [137]	Single-cell RNA-seq and transcriptomic classification study	40 patients (scRNA-seq)	Unspecified	Single-cell RNA sequencing; bulk transcriptome; consensus clustering; ML-based biomarker selection; qPCR and IHC validation	Identified 4 molecular subtypes (quiescent, macrophage-dominant, mitochondria-dominant, mixed); validated 5 biomarkers (BNIP3, FAHD1, UNG, etc.); mitochondrial dysfunction linked to immune regulation and periodontitis subtyping.

## Data Availability

Not applicable.

## Abbreviations

HR	Host response
DEGs	Differentially Expressed Genes
EDR	Electronic dental records
GCF	Gingival crevicular fluid
PD	Periodontal disease
PM	Precision medicine
PP	Precision periodontics
PPHCC	Precision Periodontal Health Care Chart
PPRA	Periodontal Risk Assessment
ROC	Receiver operating characteristic
SPR-POF	Surface plasmon resonance–plastic optical fiber
WGCNA	Weighted gene co-expression network analysis
